# Effects of ezetimibe add-on therapy for high-risk patients with dyslipidemia

**DOI:** 10.1186/1476-511X-8-41

**Published:** 2009-10-12

**Authors:** Minako Yamaoka-Tojo, Taiki Tojo, Rie Kosugi, Yuko Hatakeyama, Yuki Yoshida, Yoji Machida, Naoyoshi Aoyama, Takashi Masuda, Tohru Izumi

**Affiliations:** 1 Department of Rehabilitation, Kitasato University School of Allied Health Sciences, 1-15-1 Kitasato, Sagamihara, Kanagawa 228-8555, Japan; 2 Department of Cardioangiology, Kitasato University School of Medicine, 1-15-1 Kitasato, Sagamihara, Kanagawa 228-8555, Japan

## Abstract

**Background:**

Ezetimibe (Zetia^®^) is a potent inhibitor of cholesterol absorption that has been approved for the treatment of hypercholesterolemia. Statin, an inhibitor of cholesterol synthesis, is the first-choice drug to reduce low-density lipoprotein-cholesterol (LDL-C) for patients with hypercholesterolemia, due to its strong effect to lower the circulating LDL-C levels. Because a high dose of statins cause concern about rhabdomyolysis, it is sometimes difficult to achieve the guideline-recommended levels of LDL-C in high-risk patients with hypercholesterolemia treated with statin monotherapy. Ezetimibe has been reported to reduce LDL-C safely with both monotherapy and combination therapy with statins.

**Results:**

To investigate the effect of ezetimibe as "add-on" therapy to statin on hypercholesterolemia, we examined biomarkers and vascular endothelial function in 14 patients with hypercholesterolemia before and after the 22-week ezetimibe add-on therapy. Ezetimibe add-on therapy reduced LDL-C by 24% compared with baseline (p < 0.005), with 13 patients (93%) reaching their LDL cholesterol goals. Of the Ezetimibe add-on therapy significantly improved not only LDL-C, high-density lipoprotein-cholesterol (HDL-C), and apolipoprotein (apo)B levels, but also reduced levels of triglyceride (TG), the ratio of LDL/HDL-C, the ratio of apoB/apoA-I, and a biomarker for oxidative stress (d-ROMs). Furthermore, ezetimibe add-on therapy improved vascular endothelial function in high-risk patients with hypercholesterolemia.

**Conclusion:**

In conclusion, ezetimibe as add-on therapy to statin might be a therapeutic good option for high-risk patients with atherosclerosis.

## Background

Atherosclerosis is the most common pathological process that leads to cardiovascular diseases, a disease of large- and medium-sized arteries that is characterized by formation of atherosclerotic plaques consisting of necrotic cores, calcified regions, accumulated modified lipids, inflamed endothelial cells, smooth muscle cells, leukocytes, and foam cells [1]. Although low-density lipoprotein (LDL) remains the most important and powerful risk factor for atherosclerosis, vascular inflammation- and oxidative stress-induced mechanisms of atherosclerosis have gained tremendous interest in the past 20 years [1-4]. LDL are susceptible to structural modifications by oxidation, particularly the small dense LDL particles. Under proatherogenic conditions, nitric oxide production from endothelial cells is reduced and the burden of reactive oxygen species (ROS) is increased [5,6].

Ezetimibe (Zetia^®^) is an epoch-making cholesterol transporter inhibitor in the small intestine to treat dyslipidemia patients with high levels of LDL-C [7]. The mechanism is absorption of both food-derived cholesterol (~25%) and bile acid-derived reabsorbed cholesterol (~75%). Generally, it is difficult to achieve the target LDL-C levels by dietary therapy alone in patients with high LDL-C. HMG-CoA (or 3-hydroxy-3-methyl-glutaryl coenzyme A) reductase inhibitor, statin, is the first choice drug to reduce LDL-C for patients with high LDL-C, in that its strong effect to be lowered the circulating LDL-C levels. Because a high dose of statins cause concern about rhabdomyolysis, it is sometimes difficult to achieve the guideline-recommended levels of LDL-C in patients with high LDL-C treated with statin monotherapy. Ezetimibe has been reported to reduce LDL-C safely with both monotherapy and combination therapy with statins [8-10]. Ezetimibe is especially expected to be effective in statin-intolerant patients with high LDL-C [11]. However, it is still unclear whether or not the LDL-C-lowering by ezetimibe is effective to suppress cardiovascular events or death [12].

In the present prospective study, we investigated the contribution of ezetimibe add-on therapy to the control of hypercholesterolemia in high-risk patients on statin monotherapy, especially focused on lipid profiles and endothelial function.

## Results

To investigate the effect of ezetimibe as "add-on" therapy to statin on hypercholesterolemia, we examined biomarkers and vascular endothelial function in 14 high-risk patients for cardiovascular disease with hypercholesterolemia (Table 1). The add-on therapy of ezetimibe to statin monotherapy was safe and effective for the management of dyslipidemia in high-risk patients. After 22 weeks, ezetimibe add-on therapy reduced LDL-C by 24% compared with baseline (p < 0.005), with 13 patients (93%) reaching their LDL cholesterol goals. Of the Ezetimibe add-on therapy significantly improved not only LDL-C, high-density lipoprotein-cholesterol (HDL-C), and apolipoprotein (apo)B levels, but also reduced levels of triglyceride (TG) and d-ROMs (Table 2).

**Table 1:** Baseline characteristics of the patients included the study

**Characteristic**		**Value**
Age (year)		62 ± 10
Sex	Female	5 (36%)
Levels of vascular risk	Coronary artery disease	11 (79%)
	Diabetes mellitus	6 (43%)
	Peripheral artery disease	1 (7%)
	Stroke	3 (21%)
	Hypertension	10 (71%)
Statins	Strong statin	9 (64%)
Co-medication	Renin-angiotensin system inhibitors	14 (100%)
	β-blockers	11 (79%)
	Aspirin	10 (71%)
	Antidiabetic agents	4 (31%)

Data were shown as mean ± SD or as percentages of the total number of patients.

**Table 2:** Lipid and other biomarkers at baseline and after the ezetimibe add-on therapy

**Biomarkers**	**Baseline**	**At 22 weeks of Ezetimibe add-on therapy**	**p-value**
Triglyceride (mg/dL)	187 ± 109	134 ± 66 (-28%)	0.010
LDL-cholesterol (mg/dL)	134 ± 20	102 ± 13 (-24%)	0.020
HDL-cholesterol (mg/dL)	52 ± 11	56 ± 10	0.612
Free fatty acid (μEq/L)	377 ± 232	379 ± 204	0.726
RLP-cholesterol (mg/dL)	9.9 ± 7.0	8.8 ± 5.3	0.188
Apolipoprotein B (mg/dL)	107 ± 17	87 ± 12 (-18%)	0.004
Apolipoprotein A-I (mg/dL)	134 ± 22	141 ± 23	0.121
MDA-LDL (mg/dL)	113 ± 28	100 ± 28 (-12%)	0.039
Glucose (mg/dL)	118 ± 24	110 ± 16	0.152
HbA1c (%)	5.9 ± 0.7	6.0 ± 0.8	0.531
hs-CRP (mg/dL)	0.11 ± 0.09	0.13 ± 0.18	0.872
dROMs (U Carr)	368 ± 47	329 ± 45 (-11%)	0.014

Data are expressed as mean ± SD or as number (%). The Student's paired *t*-test was used for pairwise comparisons between values before and after administration of ezetimibe for 22 weeeks (n = 14). RLP, remnant lipoprotein; MDA, malondialdehyde-modified; hs-CRP, high-sensitivity C reactive protein; dROMs, derivatives of the reactive oxidative metabolites.

### Effects of ezetimibe on the ratio of LDL/HDL cholesterol in patients with hypercholesterolemia

**Figure 1:**
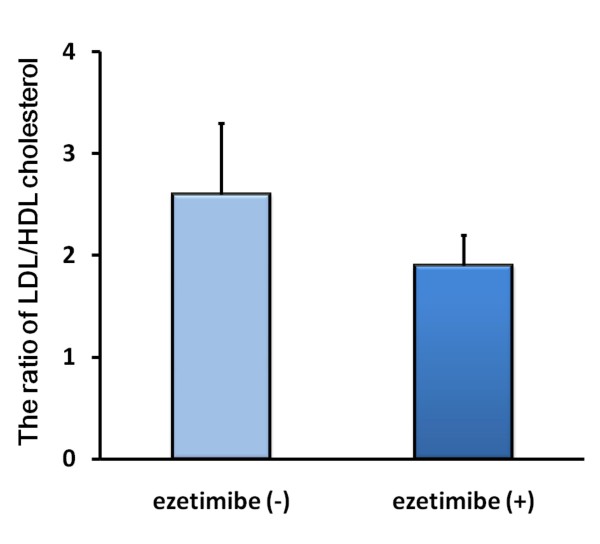
**Ezetimibe improved LDL/HDL-cholesterol ratio in patients with hypercholesterolemia**. Ezetimibe improved LDL/HDL-cholesterol ratio in patients with hypercholesterolemia. LDL- and HDL-cholesterol were measured before and after 22-week ezetimibe administration in patients with atherosclerosis-prone hypercholesterolemia (n = 14). Bars represent the mean value of the ratio ± SD.

**Figure 2:**
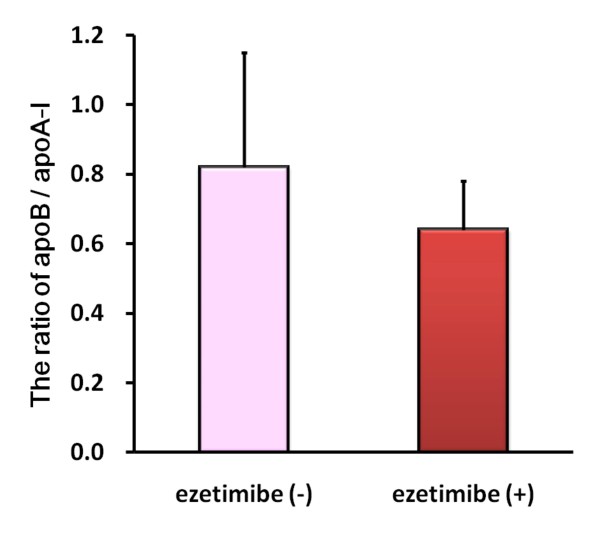
**Ezetimibe improved apolipoprotein B (apoB) to apolipoprotein A-I (apoA-I) ratio in patients with hypercholesterolemia**. Ezetimibe improved apoB to apoA-I ratio in patients with hypercholesterolemia. apoB and apoA were measured before and after 22-week ezetimibe administration in patients with atherosclerosis-prone hypercholesterolemia (n = 14). Bars represent the mean value of the ratio ± SD.

To determine atheroprotective effects of ezetimibe, we evaluated ratios of proatherogemic to antiatherogenic lipoprotein measurements, LDL-C to HDL-C ratio (LDL/HDL-C) and apoB to apoA-I ratio (apoB/A-I). As shown in Figures 1 and 2, ezetimibe improved the ratio of LDL/HDL-C and apoB/A-I. The most impressive data is that ezetimibe improved the ratio of LDL/HDL-C (by 28%; from 2.6 to 1.9; p = 0.0037) as shown in Figure 1. Furthermore, ezetimibe add-on therapy significantly reduced the ratio of apoB/A-I in high-risk patients with hypercholesterolemia (by 22%; from 0.8 to 0.6; p = 0.0052) as shown in Figure 2,.

### Effects of ezetimibe on a biomarker for oxidative stress in patients with hypercholesterolemia

**Figure 3:**
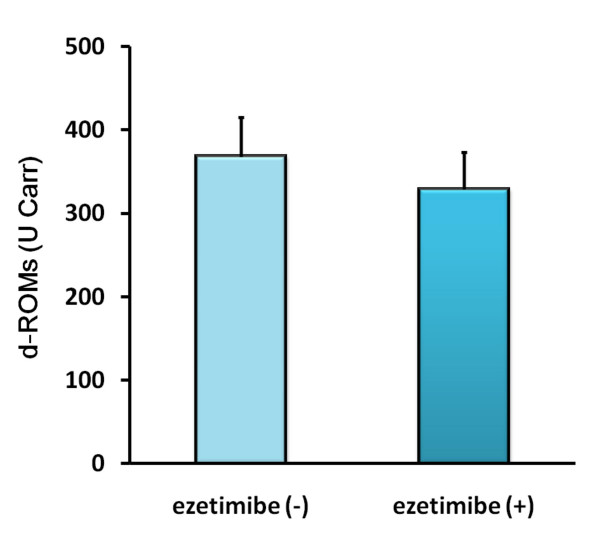
**Ezetimibe reduced a circulating oxidative marker, derivatives of reactive oxidative metabolites (d-ROMs), in patients with atherosclerosis-prone with hypercholesterolemia**. Measurements of d-ROMs were assessed before and after 22-week ezetimibe administration in patients with atherosclerosis-prone hypercholesterolemia (n = 14). Bars represent the mean value of the ratio ± SD.

A large numbers of literature has linked oxidative stress with hypertension and atherosclerosis [13]. To examine whether ezetimibe improves oxidative stress or not, we measured the derivatives of reactive oxidative metabolites (d-ROMs) test as an oxidative biomarker in patients with hypercholesterolemia before and after the ezetimibe add-on therapy with a statin (Table 2). As shown in Figure 3, the ezetimibe add-on therapy remarkably reduced d-ROMs levels in high-risk patients with hypercholesterolemia (11% reduction, p = 0.014).

### Effects of ezetimibe on vascular endothelial function in patients with hypercholesterolemia

**Figure 4:**
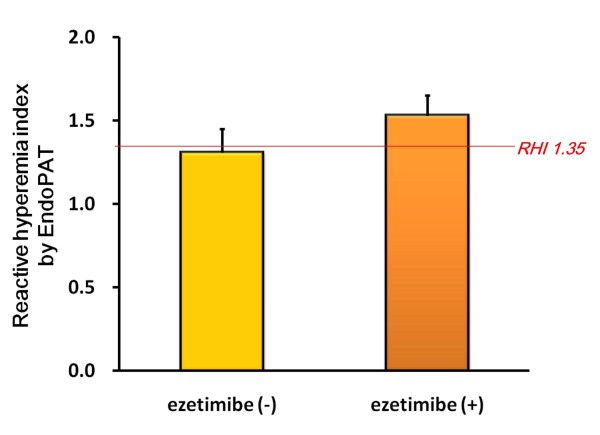
**Ezetimibe improved reactive hyperemia index (RHI) by EndoPAT™ in patients with hypercholesterolemia**. RHI were measured before and after 22-week ezetimibe administration in patients with hypercholesterolemia (n = 14). According to Bonetti P.O., et al, [14], an RHI < 1.35 was found to have a sensitivity of 80% and a specificity of 85% to identify patients with coronary endothelial dysfunction. Bars represent the mean value of the ratio ± SD.

Vascular endothelial dysfunction has been considered as the first step of atherosclerosis, we examined endothelial function using a fingertip peripheral arterial tonometry device system, EndoPAT™ (Itamar Medical Ltd., Caesarea, Israel), as a surrogate marker for cardiovascular events. After 22 weeks of ezetimibe add-on combination therapy with a statin, reactive hyperemia index (RHI) measured by the EndoPAT™ was improved in high-risk patients with hypercholesterolemia (Figure 4). Overall, there was a 14% increase in RHI at the 22nd week (from 1.31 to 1.53; p = 0.020). According to a literature [14], an RHI < 1.35 was found to have a sensitivity of 80% and a specificity of 85% to identify patients with coronary endothelial dysfunction.

## Discussion

Our results suggest that the add-on therapy of ezetimibe to statin monotherapy is safe and effective for the management of dyslipidemia in high-risk patients. In the present study, we clearly showed that ezetimibe add-on therapy reduced levels of LDL-C and apoB as well as TG and d-ROMs.

Ezetimibe, a novel lipid-lowering agent, selectively inhibits intestinal cholesterol absorption, reducing total cholesterol and TG levels and also reducing the development of atherosclerosis in apoE knockout mice [15,16]. A sterol transporter, Niemann-Pick C1-Like 1 (NPC1L1) is involved in subcellular cholesterol trafficking and plays a critical role in the absorption of intestinal cholesterol [17,18]. NPC1L1-deficient mice exhibit a substantial reduction in absorbed cholesterol, on which ezetimibe had no effect [18]. Thereafter, the molecular target of ezetimibe was revealed to be NPC1L1, which is a critical mediator of cholesterol absorption and an essential component of ezetimibe-sensitive pathway [16].

The most impressive data is that ezetimibe improved the ratio of LDL/HDL-C (p < 0.005) as shown in Figure 1. According to a recent literature, the lipid ratio of LDL/HDL-C is better monitoring predictors than single standard lipids including total cholesterol, LDL-C, and HDL-C [19]. As for initial risk measurements, several previous cohort studies and a meta-analysis study suggest that the ratio of LDL/HDL-C also have greater independent predictive values for coronary heart disease than individual serum total cholesterol or LDL-cholesterol levels [20-24]. In the current therapy for hypercholesterolemia, ezetimibe may be the most powerful agent to improve the LDL/HDL ratio compared to any statin monotherapy. Furthermore, ezetimibe add-on therapy significantly reduced the ratio of apoB to apoA-I in high-risk patients with hypercholesterolemia (p < 0.01, Figure 2). Patients who were treated adequately according to current guidelines (i.e., LDL cholesterol ≤ 100 mg/dL) still had residual major cardiovascular event risks that could be recognized by the evaluation of levels of non-HDL cholesterol or apoB [22]. On-treatment levels of non-HDL cholesterol and apoB are considered to be more closely associated with cardiovascular outcome than levels of LDL cholesterol [25]. These data suggest that ezetimibe not only reduced serum levels of LDL cholesterol but also improve total lipid profiles.

A large number of reports in the literature has linked oxidative stress with hypertension and atherosclerosis [13]. The ezetimibe add-on therapy remarkably reduced an oxidative stress marker in high-risk patients with hypercholesterolemia (Figure 3). Considering that ezetimibe monotherapy or a combination therapy with simvastatin decreased LDL tendency to peroxidation, ezetimibe may have favorable pleiotropic effects beyond the LDL-cholesterol lowering [26,27]. These data suggest that ezetimibe could contribute to atheroprotective properties through effective antioxidant actions. Since oxidized LDL induces expression of a nicotinamide adenine dinucleotide phosphate (NADPH) oxidase, gp91phox, and ROS generation in vascular endothelial cells [28], ezetimibe may attenuate ROS production through reduction of circulating ox-LDL in patients with hypercholesterolemia.

Endothelial dysfunction is the initial step of atherosclerosis. A recent report demonstrated that ezetimibe treatment attenuated vascular functions, such as endothelial dysfunction, oxidative stress, and inflammation in high-fat fed apoE-deficient mice [29]. Ezetimibe treatment markedly inhibited the development of lipid-rich plaque and also significantly improved endothelial dysfunction assessed by the vasodilator response to acetylcholine [30], accompanied by inhibition of interleukin-6 mRNA and an increase in endothelial nitric oxide synthase mRNA in the aorta. Furthermore, ezetimibe suppressed ROS generation and the ubiquitination-proteasome system in the aorta. In the present study, ezetimibe add-on therapy achieved effective RHI-improvement in high-risk patients with hypercholesterolemia (p < 0.05, Figure 4). To our knowledge, this is the first clinical data about ezetimibe-improving vascular endothelial function detected by EndoPAT.

**Figure 5:**
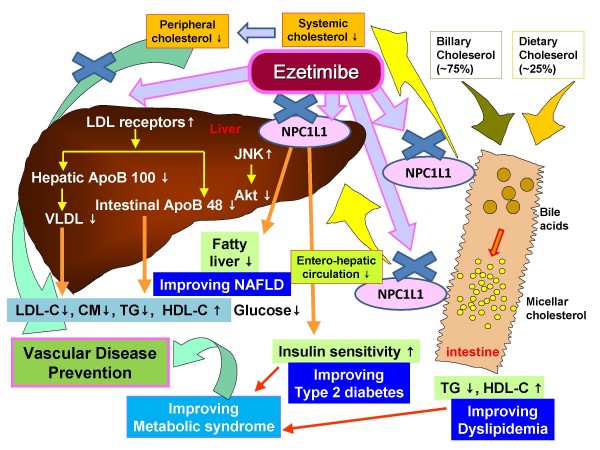
**Schematic diagram of the proposed mechanisms of ezetimibe-induced atheroprotective effects**. LDL-C, low-density lipoprotein-cholesterol; ox-LDL, oxidized LDL; VEGF, vascular endothelial growth factor; LPS, lipopolysaccharide; MCP-1, monocyte chemotactic protein-1; CM, chylomicrons; TG, triglyceride; HDL-C, high-density lipoprotein-cholesterol; NAFLD, non-alcoholic fatty liver disease; NPC1L1, Nieman-Pick C1-like 1; apoB, apolipoprotein B; VLDL, very-low density lipoprotein; JNK, Janus-family tyrosine kinase.

NPC1L1 is widely expressed in many human tissues, with the highest expression in the small intestine as well as in the liver [31,32]. Therefore, ezetimibe inhibits cholesterol absorption in the small intestine, reduces enterohepatic circulation of cholesterol, reduces uptake of free cholesterol in hepatocytes, and should affect metabolic pathways in the liver [33]. Ezetimibe improved hepatic insulin signaling as well as hepatic steatosis in Zucker Obese fatty (ZOF) rats. Ezetimibe also restored insulin sensitivity in steatotic hepatocytes *in vitro *by a reduction in hepatic ROS generation, Janus-family tyrosine kinase (JNK) activation, and endoplasmic reticulum stress. In addition, ezetimibe recovered insulin-induced Akt activation, and reduced gluconeogenic genesin the liver of ZOF rats and cultured steatotic hepatocytes [33]. Other studies have shown that ROS-dependent activation of JNK plays a role in the developing insulin resistance [34-36]. In recent clinical studies, ezetimibe treatment has been considered as an effective therapeutic option for non-alcoholic fatty liver disease (NAFLD) [31,37,38]. Several reports have concluded that ezetimibe monotherapy not only protects against high fat diet-induced dyslipidemia but also attenuates liver steatosis in an experimental NAFLD model [39,40]. In apoE knockout mice, liver weight was significantly decreased and lipid accumulation in the liver was also dramatically inhibited in the ezetimibe-treated group [29]. These accumulating data suggest that the inhibition of NPC1L1-dependent cholesterol uptake by ezetimibe may be a suitable therapeutic target for treatment of not only hypercholesterolemia but also broader aspects of metabolic disorders in patients with type 2 diabetes and/or metabolic syndrome (Figure 5).

## Conclusion

Our results suggest that ezetimibe improves lipid profiles, reduces oxidative stress, and improve endothelial function in high-risk patients with dyslipidemia, which may contribute to prevention atherosclerosis in vasculature. As an atheroprotective drug, ezetimibe may be a suitable therapeutic target for treatment of not only hypercholesterolemia but also broader aspects of metabolic disorders in patients with type 2 diabetes or metabolic syndrome or both.

## Methods

### Subjects

The study included 14 Japanese high-risk patients (36% female) with coronary artery disease or equivalents treated with statin monotherapy in the Department of Cardioangiology, Kitasato University Hospital. No patients achieved the guideline-recommended levels of LDL-C [41,42], concretely, LDL-C < 100 mg/dL for coronary artery disease patients and < 120 mg/dL for high-risk patients with diabetes, cerebrovascular disease, and/or peripheral arterial occlusive disease. Ezetimibe from Bayer Co., Ltd. (Osaka, Japan) and Schering-Plough K.K. (Tokyo, Japan) was administered once a day (10 mg/day) to all patients for 22 weeks. Lipid profiles, biochemical parameters, and adverse effects were monitored every 4-8 week through the study. None of the patients took any other drugs and/or any advice for diet and lifestyle modification that affect lipid profile or lipid metabolism. Except the ezetimibe add-on therapy, every medication was continued without any dose changes during the study period. Total calorie intake and composition of the diet were kept constant for each patient.

All subjects gave written informed consent before participating in this study, and the ethics committee of the Kitasato University Hospital approved the study design.

### Measurement of serum sample

Fasting blood samples were taken from each of the 14 enrolled patients before and after the ezetimibe add-on study. Serum was centrifuged (1500 g for 15 min at 4°C) and stored at 4°C until measurement within couple days for biochemical markers, such as TG, LDL-C, HDL-C, alanine aminotransferase, aspartate aminotransferase, serum creatinine, creatine kinase, free fatty acid, remnant lipoprotein-cholesterol, apoB, apoA-I, malondialdehyde-modified-LDL, glucose, HbA1c, and high-sensitivity C reactive protein.

### Measurement of d-ROMs

Serum was centrifuged (1500 g for 15 min at 4°C) and stored at -20°C until measurement within one week for free oxygen radical monitor, d-ROMs. The d-ROMs were evaluated spectrophotometrically by Free Radical Analytical System (FRAS4^®^, Diacron International, Grosseto, Italy) [43,44]. The d-ROMs test is based on the ability of transition metals to catalyze, in the presence of peroxides, the formation of free radicals which are then trapped by an alchylamine. The alchylamine reacts forming a colored radical detectable at 505 nm through a kinetic reaction which is linear up to 500 U Carr (Carratelli units). The determination of free radicals can be made with a normal spectrophotometer. The normal range has been determined as 250-300 U Carr [43].

### Measurement of vascular endothelial function by EndoPAT

Vascular endothelial function was examined reactive hyperemia by EndoPAT™ [45] in patients with hypercholesterolemia. Using a fingertip peripheral arterial tonometry device, we measured digital pulse amplitude in patients with hypercholesterolemia for 5 minutes at baseline and after a reactive hyperemia induced by a 5-minute forearm cuff occlusion. RHI was measured before and after 22-week ezetimibe administration in patients with hypercholesterolemia.

### Statistical analysis

The results were expressed as mean ± SD. The Student's paired *t*-test was used for patients between values before and after ezetimibe administration. A value of p < 0.05 was considered to be statistically significant.

### Competing interests

The authors declare that they have no competing interests.

## Authors' contributions

MY-T participated in the design of the study and performed the statistical analysis. TT conceived of the study, and participated in its design and coordination and helped to draft the manuscript. Other authors participated in enrolling patients in the study. All authors read and approved the final manuscript.
